# Clinical and Paraclinical Biomarkers and the Hitches to Assess Conversion to Secondary Progressive Multiple Sclerosis: A Systematic Review

**DOI:** 10.3389/fneur.2021.666868

**Published:** 2021-08-26

**Authors:** Nik Krajnc, Gabriel Bsteh, Thomas Berger

**Affiliations:** ^1^Department of Neurology, Medical University of Vienna, Vienna, Austria; ^2^Department of Neurology, University Medical Centre Ljubljana, Ljubljana, Slovenia

**Keywords:** multiple sclerosis, progression, neurodegeneration, biomarkers, brain atrophy, neurofilaments, optical coherence tomography

## Abstract

Conversion to secondary progressive (SP) course is the decisive factor for long-term prognosis in relapsing multiple sclerosis (MS), generally considered the clinical equivalent of progressive MS-associated neuroaxonal degeneration. Evidence is accumulating that both inflammation and neurodegeneration are present along a continuum of pathologic processes in all phases of MS. While inflammation is the prominent feature in early stages, its quality changes and relative importance to disease course decreases while neurodegenerative processes prevail with ongoing disease. Consequently, anti-inflammatory disease-modifying therapies successfully used in relapsing MS are ineffective in SPMS, whereas specific treatment for the latter is increasingly a focus of MS research. Therefore, the prevention, but also the (anticipatory) diagnosis of SPMS, is of crucial importance. The problem is that currently SPMS diagnosis is exclusively based on retrospectively assessing the increase of overt physical disability usually over the past 6–12 months. This inevitably results in a delay of diagnosis of up to 3 years resulting in periods of uncertainty and, thus, making early therapy adaptation to prevent SPMS conversion impossible. Hence, there is an urgent need for reliable and objective biomarkers to prospectively predict and define SPMS conversion. Here, we review current evidence on clinical parameters, magnetic resonance imaging and optical coherence tomography measures, and serum and cerebrospinal fluid biomarkers in the context of MS-associated neurodegeneration and SPMS conversion. Ultimately, we discuss the necessity of multimodal approaches in order to approach objective definition and prediction of conversion to SPMS.

## Introduction

Multiple sclerosis (MS) is an autoimmune demyelinating disease affecting the central nervous system, which results from the interaction of genetic and environmental factors that remain only partially understood (1, 2). The majority of patients (85%) initially follow a relapsing-remitting course (RRMS), defined by acute exacerbations and periods of relative clinical stability in between (3). In ~10–15%, patients suffer from a progressive decline in neurological function, called primary progressive multiple sclerosis (PPMS) (4, 5). Natural history of RRMS encompasses conversion to a secondary progressive course (SPMS), which is a gradual process characterized by irreversible disability progression, independent of relapses (6). SPMS conversion is the decisive factor for long-term prognosis in MS, generally considered the clinical equivalent of progressive MS-associated neuroaxonal degeneration with limited and qualitatively changed inflammatory ability (7–10). Although once nearly 10% of patients with RRMS converted to SPMS within 5 years, 25% in 10 years, and 75% in 30 years, the 10-, 15-, and 20-year risk of conversion to SPMS seems to be much lower nowadays (2, 9, and 27%, respectively), due to earlier diagnosis and possible treatment options (11–13). However, anti-inflammatory disease-modifying interval therapies (DMT) successfully used in RRMS are ineffective in SPMS (without superimposed relapses), whereas specific treatment for SPMS is increasingly a focus of MS research. Therefore, the prevention, but also the (anticipatory) diagnosis of SPMS, is of crucial importance.

The problem is that SPMS diagnosis is yet exclusively based on retrospectively assessing the increase of overt physical disability usually over the past 6–12 months. Currently, the most widely used definition of SPMS is the occurrence of disability progression of ≥1 Expanded Disability Status Scale (EDSS) steps (when the EDSS score ≤ 5.5) or ≥0.5 EDSS steps (when the EDSS score ≥6) in the absence of a relapse, and a minimum EDSS score of 4 and pyramidal functional system score of 2 (14). Besides, the inflammatory and neurodegenerative process that have been once thought to be almost strictly separated in relapsing and progressive MS, respectively, are now thought to be a part of a continuum, in which inflammatory activity prevails at the beginning of the disease, but can occur in the later stages of the disease, too—and vice versa. Recent studies have confirmed that disability progression can be seen even in patients with RRMS in the absence of relapses (15, 16). This “silent progression” is associated with brain and retinal atrophy, and suggests that neurodegeneration, which is the driving mechanism of disability progression in patients with SPMS, likely begins much sooner than generally recognized (17, 18).

Defining SPMS retrospectively inevitably results in a delay of diagnosis, reportedly of up to 3 years, resulting in periods of uncertainty and, thus, also making early therapy adaptation (including timely escalation) to prevent SPMS conversion impossible (19, 20). Hence, there is an urgent need for reliable and objective biomarkers to prospectively predict and define SPMS conversion.

An objective definition and reliable prediction of SPMS conversion is especially important in an era in which new therapies with potential neuroprotective effects are introduced. In this way, late RRMS and early SPMS may represent a window of opportunity for intervention to delay or even prevent disability progression. Therefore, the primary objective of this systematic review was to assess the role of potential clinical and paraclinical biomarkers to determine conversion to SPMS.

## Methods

### Search Methods

A review of the literature concerning biomarkers in secondary progressive MS was performed using PubMed with no restriction placed on country or publication date. Search terms included the following: biomarkers, Expanded Disability Status Scale, Multiple Sclerosis Functional Composite, Symbol Digit Modalities Test, Low-Contrast Letter Acuity, olfactory function, magnetic resonance imaging, brain atrophy, slowly expanding lesion, spinal cord atrophy, optical coherence tomography, peripapillary retinal nerve fiber layer, macular ganglion cell-inner plexiform layer, neurofilaments, glial fibrillary acidic protein, soluble triggering receptor 2, OR chitinase 3-like 1 AND disease progression AND multiple sclerosis. Relevant articles were also found by scanning the references of found articles (backward search) and locating newer articles that included the original cited paper (forward search). The last search was performed on the October 31, 2020. The search yielded 4,508 articles.

### Selection Criteria

**Figure 1:**
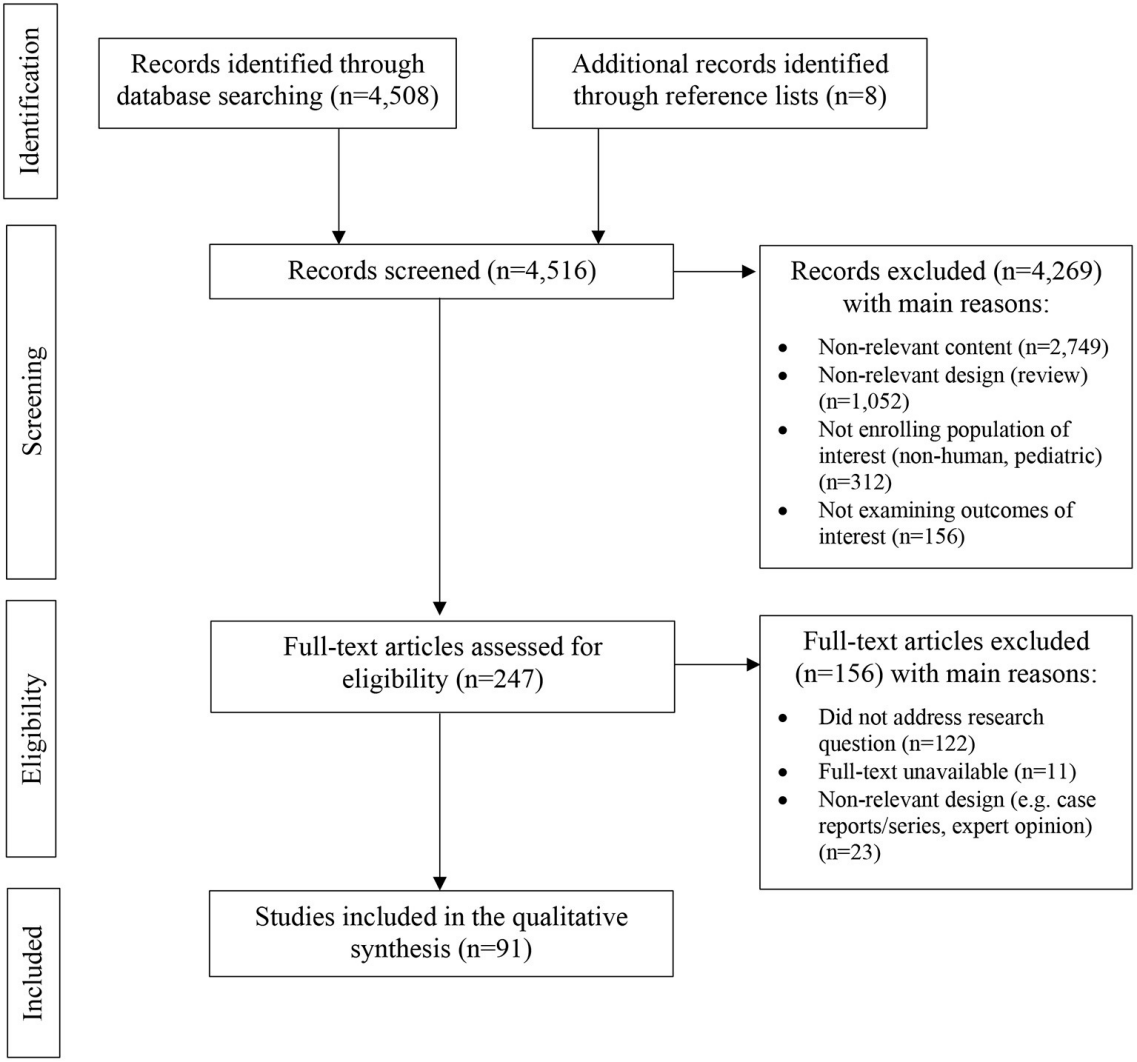
PRISMA flow diagram demonstrating included and excluded studies, and the reasons for exclusion in the systematic review.

Our selection criteria were language (English), focus of the study (to determine the progression of MS), and an original contribution of the publication, regardless of the interventional or non-interventional nature of the study. Data from reports were extracted from each report separately. Case reports were excluded with an exception of one case report presented with qualitative data. After selection criteria were applied, 4,261 articles were excluded. We found 247 eligible articles, among which 91 were included in the review (Table 1, Figure 1).

**Table 1:** Number of articles after applying selection criteria for each biomarker of conversion to secondary progressive multiple sclerosis (SPMS).

**Biomarkers of conversion to SPMS**		**Eligible**	**Articles included in**
		**articles (*n*)**	**the review (*n*)**
Clinical biomarkers	EDSS	38	6
	MSFC	18	12
	SDMT	16	11
	Visual function	4	3
	Olfactory function	12	7
MRI	Brain atrophy	49	9
	SELs	13	7
	Spinal cord atrophy	22	8
OCT	pRNFL, mGCIPL	15	11
Biomarkers in blood	Nf	21	8
and CSF	GFAP	11	4
	sTREM2	2	1
	CHI3L1	11	4

*CHI3L1, chitinase 3-like 1; CSF, cerebrospinal fluid; EDSS, Expanded Disability Status Scale; GFAP, glial fibrillary acidic protein; mGCIPL, macular ganglion cell-inner plexiform layer; MRI, magnetic resonance imaging; MSFC, Multiple Sclerosis Functional Composite; Nf, neurofilament; OCT, optical coherence tomography; pRNFL, peripapillary retinal nerve fiber layer; SDMT, Symbol Digit Modalities Test; SELs, slowly expanding lesions; sTREM2, soluble triggering receptor 2*.

### Evidence Grading Process

The methodological quality of the studies included in this review was graded using the Grades of Recommendation, Assessment, Development, and Evaluation (GRADE) tool for best-evidence synthesis (Table 2). The systematic review was prepared according to the latest PRISMA (Preferred Reporting Items for Systematic Reviews and Meta-Analyses) guidelines.

**Table 2:** The clinical and paraclinical biomarkers of conversion to secondary progressive multiple sclerosis (SPMS).

**Biomarkers of conversion**		**Measure**	**Pathophysiological**	**Advantages**	**Disadvantages**	**Evidence**
**to SPMS**			**correlate**			**grade**
Clinical biomarkers	EDSS	20 steps from 0 to 10 Relevant increase: ≥1 point when score is ≤ 5.5, ≥0.5 when score is ≥6.0	Neuroaxonal damage, primarily spinal	Easily accessible Time efficient	Depends on walking ability Does not reflect cognition Lacks inter- and intrarater reliability	High
	MSFC	Time to walk 25 feet (T25FW) and put nine pegs in and out of a box with holes (9HPT), number of correct out of 60 possible answers (PASAT) Relevant change: ≥20% in MSFC subscores	Neuroaxonal damage, primarily cerebral	Easily accessible Evaluates measures not included in EDSS	PASAT less sensitive to detect cognitive worsening 9HPT and PASAT demonstrate practice effect	High
	SDMT	Number of correct substitutions within a 90 s interval (maximum 110) Relevant change: ≥4 points or ≥10%	Neuroaxonal damage, primarily cortical and subcortical	Time efficient Easy to administer Change sensitive Independent of language	Practice effect	High
	Visual function	Number of correctly identified letters (LCLA chart) Relevant change: ≥7 letters loss	Neuroaxonal damage in anterior visual pathway	Time efficient	Requires a retroilluminated cabinet or a standardized room	Low
	Olfactory function	Number of correctly discriminated (D) and identified (I) odors DI-score of maximum 32 points Relevant change: ≥2 points	Neuroaxonal damage in olfaction-related brain regions	Time efficient Easy to administer Easily accessible	Multiple external confounders (smoking, hunger state, upper respiratory tract infection, corticosteroids)	Low
MRI	Brain atrophy	Global and regional cortical and subcortical atrophy Relevant change: ≥0.4% per year	Neuroaxonal damage, cerebral	Highly reproducible	Pseudoatrophy effect Dependent on confounding factors (hydration, diurnal fluctuations, lifestyle, comorbidities) Technical limitations (heterogenous acquisition protocol, scanner variability)	Moderate
	SELs	Number of iron rim lesions Relevant change: not known	Chronic demyelination, leading to neuroaxonal damage	*In vivo* assessment of chronic demyelination Highly reproducible	Technical limitations	Low
	Spinal cord atrophy	Cervical spinal cord average CSA Relevant change: not known	Neuroaxonal damage, spinal cord	Higher rate of change compared to brain atrophy	Anatomical (high mobility, low dimensions) and imaging (low tissue contrast) limitations High impact of lesions on measurements	Moderate
OCT	pRNFL	Thickness in μm Relevant change: >1.5 μm	Axonal degeneration, antero- and retrograde	Non-invasive Easily accessible Highly reproducible	Prone to confounding from optic neuritis	Moderate
	GCIPL	Thickness in μm Relevant change: >1.0 μm	Neuronal degeneration	Faster detection of damage, larger range of change and less prone to confounding from optic neuritis (compared to pRNFL) Higher sensitivity (compared to brain atrophy)	Requires rigorous quality control for image quality and segmentation	Moderate
Biomarkers in blood and CSF	Nf	Nf levels in serum and/or CSF Relevant change: not known	Neuroaxonal degeneration	Blood: Quick and easy collection Easily available for repeated measurement	CSF: invasive, low availability for repeated measurement low accessibility of Simoa	Moderate
	GFAP	GFAP levels in serum and/or CSF Relevant change: not known	Reactive astrogliosis			Low
	sTREM2	sTREM2 levels in serum and/or CSF Relevant change: not known	Microglial activation			Low
	CHI3L1	CHI3L1 levels in serum and/or CSF Relevant change: not known	Reactive astrogliosis			Low

*CHI3L1, chitinase 3-like 1; CSA, cross-sectional area; CSF, cerebrospinal fluid; EDSS, Expanded Disability Status Scale; GFAP, glial fibrillary acidic protein; LCLA, low-contrast letter acuity; mGCIPL, macular ganglion cell-inner plexiform layer; MRI, magnetic resonance imaging; MSFC, Multiple Sclerosis Functional Composite; Nf, neurofilament; OCT, optical coherence tomography; ON, optic neuritis; PASAT, Paced Auditory Serial Addition Test; pRNFL, peripapill ary retinal nerve fiber layer; SDMT, Symbol Digit Modalities Test; SELs, slowly expanding lesions; sTREM2, soluble triggering receptor 2; T25FW, Timed 25-Foot Walk Test; 9HPT, 9-Hole Peg Test*.

## Clinical Parameters

### EDSS

In most clinical trials, EDSS has been and is used to measure disability progression. It consists of 20 steps ranging from 0 to 10, assessing MS-related impairment based on neurological examination (EDSS score <4.0), walking ability (EDSS score 4.0–6.0), or other functional impairments (EDSS score ≥6.5) (21). However, it has proven less sensitive in detecting all clinically relevant contributors to disability progression in SPMS patients, especially upper-extremity and cognitive dysfunction (22, 23). Besides that, the mid-range of the EDSS overvalues long-distance ambulation and lacks inter- and intrarater reliability (24–26). In the London (Ontario) cohort, the median time from EDSS score 6.0 to 8.0 was 7.9 years, arguing against using the EDSS as the primary outcome in trials in which more disabled patients with SPMS are included (27).

### MSFC

The Multiple Sclerosis Functional Composite (MSFC) is a composite score assessing short-distance ambulation [Timed 25-Foot Walk Test (T25FW)], upper-extremity function [9-Hole Peg Test (9HPT)], and cognitive function [Paced Auditory Serial Addition Test (PASAT)].

T25FW and 9HPT measure time to walk 25 feet and to put nine pegs in and out of a box with holes, respectively. They are able to identify disability progression in SPMS patients more frequently than EDSS (28, 29). The threshold for T25FW and 9HPT to detect clinically meaningful disability progression is reported at 20% or more (30–32). Still, the PASAT, a test of auditory information processing speed, flexibility, and calculation ability, has not been shown to sensitively detect cognitive worsening in SPMS (33, 34). As the 9HPT and PASAT have well-demonstrated practice effects, meaning that participants learn how to perform the test and improve their scores with each repetition, the T25FW seems to be the most reliable clinical test to sense disease progression (35, 36).

In the IMPACT (The MS Progressive Avonex Clinical Trial) and ASCEND (Effect of Natalizumab on Disease Progression in Secondary Progressive Multiple Sclerosis) clinical trials, evaluating the efficacy of intramuscular interferon beta 1a and natalizumab treatment in SPMS, respectively, the T25FW was the single outcome measure with the greatest proportion of patients showing disability progression (37, 38). While the 9HTP also shows a small but significant rate of change over time, it is more prone to fluctuations (39). The EDSS-Plus composite score (EDSS, T25FW, and 9HPT) is roughly twice as sensitive as EDSS alone in detecting disability progression in SPMS patients (59.5 vs. 24.7%, respectively) (30). With EDSS 3.0–6.5, and a T25FW of 8 s or more, the progression rate above 40% was met in both clinical trials (40). However, although the focus of the clinical trials mentioned was disability progression in already diagnosed SPMS rather than the conversion from RRMS to SPMS, a composite score could be used as a sensitive biomarker in determining the conversion to SPMS.

### SDMT

Beside physical impairment, 40–65% of patients with progressive MS have some degree of cognitive impairment (41). Cognitive function correlates even closer with quality of life than the measures of physical impairment but is frequently underestimated when only EDSS is used (42). The areas most affected comprise information processing speed, complex attention, memory, and executive function (43). The cognitive impairment is correlated with the atrophy of cortical and subcortical areas, the corpus callosum, and the superior longitudinal fasciculus (44, 45).

With a specificity of 60% and a sensitivity of 91%, the Symbol Digit Modalities Test (SDMT) presents a sentinel test for cognitive impairment in patients with MS (46, 47). It evaluates the sustained attention, the capacity of concentration, and the visuomotor speed. When compared to the PASAT, the SDMT proved superior with a higher sensitivity (48–50). In general, a four-point or 10% change in SDMT is considered clinically relevant (51). It correlates less strongly with EDSS and the other performance measures (T25FW, 9HPT), providing additional information by assessing the function not captured by the other measures (52). Among different neuropsychological and language performances, the SDMT showed to have the greatest effect size between RRMS and SPMS (53). In this respect, the SDMT is used as the primary endpoint to assess cognitive changes in patients with SPMS, e.g., in the AMASIA study [Impact of Mayzent® (Siponimod) on Secondary Progressive Multiple Sclerosis Patients in a Long-Term Non-Interventional Study in Germany] (54). However, it displays a significant practice effect when brief inter-assessment intervals are used, which becomes less pronounced with the progression of the disease (55, 56). Therefore, a change in key is proposed to make the interpretation of the results less biased. Still, a recent study suggested that SDMT scores improve throughout follow-up, possibly due to a practice effect, and in that way does not reflect the steady cognitive decline that patients with SPMS experience (57).

### Assessment of Visual Function

The anterior visual pathway is affected in more than 90% of MS patients over the course of the disease (58). Among a variety of available measures of visual function, visual contrast threshold is the most promising in MS, defined as the minimum amount of contrast necessary for an individual to discern an object from its background. Visual contrast is assessed by Sloan low-contrast letter acuity (LCLA) charts, which are based on Early Treatment Diabetic Retinopathy Study high-contrast visual acuity (HCLA) charts, but using gray letters with 2.5 and 1.25% contrast level as opposed to black letters (100% contrast level) (59). The charts provide a continuous measure with seven letters of LCLA loss considered to be meaningful and beyond the threshold of test-retest variability (60).

LCLA has been found to be altered in patients with MS, even when HCLA appears normal (61). It shows good structure-function-correlation both with retinal atrophy and lesions in the posterior visual pathway (62–64). Therefore, there have been calls for the inclusion of LCLA in MSFC to reflect visual function (49, 65). The IMPACT study was the first clinical trial to use LCLA as an exploratory visual outcome, reporting good correlation with disability, MSFC and EDSS (65, 66). Recently, progressive visual impairment has been proposed as an additional modality in defining SPMS conversion in a case report (67).

### Assessment of Olfactory Function

Impairment of olfactory function is an increasingly recognized feature of MS with different modalities reflecting different aspects of MS pathology (68–70). The capacity to correctly identify odors (identification) and discriminate them (discrimination) is predominantly affected in progressive and more advanced MS (69). It slowly deteriorates over time in association with progressing physical disability (68). In contrast, olfactory threshold is impaired in early, active MS, and predicts short-term inflammatory disease activity (71–73).

The sum score of discrimination and identification (DI score) has been shown to correlate with disease duration, physical disability, reduced cognitive function, and reduced retinal thickness of MS patients (69, 74). Moreover, a recent study has shown a robust correlation between impairment of DI score and decreased gray matter concentration in the putamen and temporomesial brain regions in MS patients (75). The olfactory dysfunction is related to several cognitive measures, including SDMT (76).

## Magnetic Resonance Imaging

### Brain Atrophy

Gray matter atrophy quantified by means of MRI volumetry is a well-established imaging marker of neurodegeneration in MS (77). It is typically measured from standard 3D T1-weighted images, using fully automated approaches, among which the SIENA method (Structural Image Evaluation of Normalized Atrophy) and Brain Boundary Shift Integral (BBSI) are most commonly used (78–81). SIENA performs segmentation of brain from non-brain tissue, estimates the outer skull surface as a normalizing factor, and aligns the two scans to correct for changes in image geometry. The registered segmented brain images are used to find local atrophy, measured on the basis of the movement of image edges (81). It has a good test–retest reliability with an error of 0.17% on an MS data set (82). Segmentation-based algorithms used in a semiautomatic way (with manual correction) are considered as reference standard techniques, but are time consuming and less reproducible (83).

Gray matter atrophy occurs in all phenotypes of MS and is associated with disability accumulation (84). Recently, cut-offs to distinguish pathological brain atrophy related to MS from the physiological change have been established, with 0.40% per year performing best for detecting physical disability progression (65% sensitivity, 80% specificity) (85). Cortical atrophy seems to accelerate in progressive MS compared to RRMS (−0.87 vs. −0.48%, respectively) (86). Some brain areas display earlier atrophy compared to others, namely, cingulate cortex, insular and temporal cortical gray matter, and the deep gray matter (putamen, caudate nucleus) (84, 87). Cortical atrophy patterns show stronger association with clinical (especially cognitive) dysfunction than global cortical atrophy (86, 87).

Thalamic volume is another MRI volumetric measure of neurodegeneration in MS. Thalamic atrophy at baseline is associated with higher risk for 5-year EDSS increase as well as for not reaching criteria of no evidence of disease activity (NEDA-3) after 2 years (88, 89). Atrophy of anterior thalamic nucleus is also associated with decreased cognitive processing speed (90). However, the rate of decline shows little variation throughout the disease at an estimated −0.71% per year (95% CI = −0.77 to −0.64%) (91). Among regional brain atrophy, corpus callosum seems to be one of the most sensitive MRI markers for memory and processing speed (92). In contrast to the rate of thalamic atrophy, a study of the MAGNIMS study group showed SPMS to have a higher rate of temporal (−1.21%) and parietal (−1.24%) gray matter atrophy. However, only the atrophy rate in the deep gray matter was associated with disability accumulation (*p* < 0.001) (84).

There are several external confounding factors that need to be taken into account when analyzing brain atrophy, including hydration changes, diurnal fluctuations, lifestyle (smoking, alcohol consumption), menstrual cycle, and comorbidities (93–97). Whereas inflammation can transiently increase brain volume in the short-term, DMT reduce edema, causing accelerated, non-tissue-related brain volume loss, known as pseudoatrophy (98). However, these confounding factors only result in minor volume changes. Besides the confounding factors, there are also technical barriers that pose a challenge in the adoption of atrophy in clinical practice. These include heterogeneity in acquisition protocol, distortion differences, and scanner variability, to name a few (99, 100).

### Slowly Expanding Lesions

In MS, some lesions remyelinate early after the demyelinating event, evolving into remyelinated shadow plaques which is protective against axonal degeneration (101, 102). While those predominate in early RRMS, some lesions develop into smoldering plaques or slowly expanding lesions (SELs) which are more prominent in progressive MS (12–28% plaques) and seem to indicate progressive disease activity (103–106). They are associated with incomplete remyelination which results in irreparable myelin loss, leading to axonal degeneration (107, 108).

Histopathologically, SELs are characterized by an inactive center with no or few macrophages, surrounded by an iron rim containing microglia/macrophages with a pro-inflammatory activation status. While most studies on SEL have been conducted on 7T MRI, which has limited availability, it has been consistently shown that SEL can also be sensitively detected on 3T MRI using several different sequences, among which the susceptibility-weighted imaging (SWI) is the most reliable (109–114). SELs expand toward the surrounding white matter in comparison with non-iron lesions, which significantly shrink over time (115). They seem to be more destructive, too, reflected by T1 hyperintensities (black holes) which are associated with greater reduction in myelin and axonal density (116–118).

Patients with multiple SELs (≥4 SELs) have more aggressive disease (higher lesion load and ventricular volumes, lower brain and basal ganglia volumes) and reach higher motor (EDSS) and cognitive disability (SDMT, PASAT) or transit to disease progression at a younger age (119, 120). Another study confirmed that SELs significantly predict clinical progression, evaluated by EDSS, T25FW, and 9HPT (121).

As edge-related iron accumulation might separate SEL from the lesions with a higher remyelination potential, SWI-based iron imaging may present a useful imaging biomarker for progressive MS.

Besides that, SELs seem to have a good imaging–pathologic correlation, which is why we think they could be used routinely to determine disease progression or even conversion to SPMS.

### Spinal Cord Atrophy

Spinal cord atrophy (SCA) is another promising biomarker of MS-associated neurodegeneration. A recently published meta-analysis has confirmed the correlation between SCA and clinical disability, assessed by EDSS (122). When comparing the cross-sectional area (CSA) of a spinal cord, it can differentiate between RRMS and progressive types of MS (*p* < 0.001) (123). SCA also progresses faster in patients exhibiting disease progression at 2 years (124). A recent study has confirmed that SPMS and RRMS patients differ in cervical spinal cord average CSA (*p* = 0.03) as well as in C7 area (*p* = 0.002) (125). Atrophy of the upper cervical cord is most evident in the antero-posterior direction, and attains a cranio-caudal pattern with the progression of the disease (126, 127). It presents a sensitive biomarker, especially as the estimated annual rate of SCA is greater when compared to the rate of brain atrophy in patients with MS (−1.78 vs. −0.5%) (123, 128).

However, assessment of the SCA is technically more difficult than brain segmentation due to anatomical (higher mobility, smaller dimensions) and imaging (lower tissue contrast) features of the spinal cord. Focal lesions also add variance to SCA measures, with acute lesions and chronic lesions causing swelling and shrinking, respectively (129). SCA is usually measured on T1-weighted gradient recalled sequences, with the mean upper cervical cord area, a measurement of CSA at the level between the C2 and C3 vertebrae, being most commonly used (130–132). A study of Liu and coworkers showed that the C2/C3 CSA is comparable to the CSA 2.5 cm below the inferior margin of pons, which may be of interest in longitudinal studies or trials where spinal cord measurements were not included as an original outcome measure, but could be obtained from the brain scans (133).

## Optical Coherence Tomography

Optical coherence tomography (OCT) is a non-invasive and accessible technique that uses near-infrared light to create images of the retina (134). It can measure the thickness of peripapillary retinal nerve fiber layer (pRNFL) and ganglion cell-inner plexiform layer (GCIPL), which are both robust indicators of neuroaxonal degeneration in MS (135). Since time domain OCT (TD-OCT) has been supplanted by spectral domain OCT (SD-OCT), which provides a better image resolution and enables the use of segmentation algorithms, validity has so much increased that small changes in the micrometer spectrum can be reliably reproduced (136). Among fully automated segmentation techniques, two commonly utilized are Cirrus and Spectralis (137). Both Spectralis and Cirrus proved to have high reproducibility and repeatability in both pRNFL and GCIPL measurements, especially when eye tracking and averaging of multiple images are used (138–140).

Several studies have shown that pRNFL and GCIPL are significantly reduced in patients with MS regardless of prior optic neuritis (ON) (136, 141, 142). They are associated with both present and future physical and cognitive disabilities as well as brain atrophy, while short-term relapse activity (apart from ON) has little or no effect on pRNFL and GCIPL thinning (74, 135, 143–145). A baseline pRNFL thickness in the eyes without prior ON of ≤ 87 μm (Cirrus) or 88 μm (Spectralis) approximately doubled the risk of disability worsening and cognitive decline during the following 2–5 years (74, 144). Similar results were found for a baseline GCIPL thickness below 77 μm (Spectralis) (146).

OCT can potentially help differentiate between MS subtypes, with RRMS patients having significantly lower RNFL thinning compared to patients with SPMS. There was no statistically significant difference between the RNFL thickness in patients with both subtypes of progressive MS (SPMS, PPMS) (147). With a specificity of 90% and a sensitivity of 76.1%, annual pRNFL thinning rate of more than 1.5 μm is able to distinguish between stable and progressive MS; the risk is increased by 15-fold (148). A recent study found that an annual loss in GCIPL above a cut-off ≥1 μm accurately identifies clinically progressing patients with 87% sensitivity and 90% specificity, and presents a strong predictor of clinical progression (146).

One of the limitations of pRNFL measurement is its dependence on optic disc swelling at the time of ON, and its reduction after the episode of ON, which makes GCIPL superior for detection of early atrophy following ON (149, 150). Besides that, RNFL is not homogenous but thicker around the optic disc, decreasing the reliability of scan acquisition (151). Progressive thinning of GCIPL, and to a lesser extent, pRNFL in absence of inflammatory episodes makes the measure a compelling model for MS associated neurodegeneration and, thus, a promising candidate biomarker for definition and prediction of conversion to SPMS.

## Biomarkers in Blood and Cerebrospinal Fluid

### Neurofilaments

Neurofilaments (Nf) are major components of the neuronal cytoskeleton and neuroaxonal damage causes their release into the extracellular space and further into the cerebrospinal fluid (CSF) and the blood. Thus, Nf have recently garnered increasing attention as a biomarker of axonal injury (152).

In MS, NfL levels in the CSF are associated with the occurrence of neurological disability, MRI lesions, and treatment status in MS (153, 154). CSF NfL levels at the time of diagnosis seem to be an early predictive biomarker of long-term clinical outcome and conversion from RRMS to SPMS (155, 156).

Until recently, Nf studies were limited to CSF as detection systems were not sensitive enough to quantitate the physiologically lower levels of Nf in the peripheral blood. This restricts clinical applicability since obtaining CSF requires lumbar puncture, which is an invasive procedure and necessitates stringent indication for diagnostic purpose (152). Also, repeated measurement is hardly feasible as repeated lumbar punctures are difficult to justify and seldomly tolerated by patients (152).

The advent of the SIngle MOlecule Array (Simoa®) technology enables highly sensitive quantitation of the Nf light (NfL) subunit in the peripheral blood (153). Importantly, several studies have demonstrated that CSF and serum NfL (sNfL) levels are highly correlated paving the road for application of NfL as biomarker available for serial measurement (152). sNfL levels correlate with disability and increase over time, even in the absence of prior/subsequent disability progression, and are associated with various MRI parameters of neuroaxonal degeneration (T1 black holes, brain and spinal cord atrophy) (157–160). However, several current limitations need to be addressed: Nf levels are age-dependent and may be confounded by certain concomitant disorders (e.g., physical activity, trauma, small vessel disease); Nf correlations are based on group-wise rather than individual evaluations (161).

Therefore, serum Nf are a promising candidate biomarker for definition and prediction of SPMS conversion, but utility in clinical routine practice awaits confirmation.

### Glial Fibrillary Acidic Protein

Glial fibrillary acidic protein (GFAP) is one of the major intermediate cytoskeletal proteins of astrocytes and presents a well-established marker of reactive astrogliosis. The upregulation of GFAP is critically important for the formation of extended and thickened astrocytic processes observed in reactive astrogliosis at the site of the injury (162). The latter is not necessarily connected with glial scar formation, and its re- or demyelinating potential depends on a number of factors, including the timing after injury, the microenvironment of the lesion, and its interaction with other cell types and factors influencing their activation (163–165). However, extensive astrocytosis leads to the formation of the astroglial scar which plays a role in the progression of MS (166).

Patients with progressive MS have significantly higher levels of GFAP in CSF compared to the patients with clinically isolated syndrome or early RRMS (167). GFAP levels in CSF and serum correlate with neurological disability (EDSS) and disease progression; the mean annual increase of GFAP is significantly higher in SPMS patients compared to RMS and correlates with sNfL and the MRI lesion count, especially in progressive MS patients (168–170).

### Soluble Triggering Receptor 2

The triggering receptor expressed on myeloid cells 2 (TREM2) is found on the cell surface of macrophages and microglia cells. Activation of TREM2 is associated with reduced tissue destruction in animal models (171). In contrast, the soluble form of TREM2 (sTREM2) detectable in CSF appears to reflect the extent of microglial activation, with elevated sTREM2 concentrations indicating increased microglial activation (170, 171).

An essential aspect of the pathophysiology of SPMS conversion is seen in a microcompartmentalization of inflammation within the central nervous system, which is primarily mediated by macrophages and microglia (172). Intriguingly, a small study has recently described an increase in sTREM2 in CSF in patients with progressive MS (173). Therefore, sTREM2 could be important as a biomarker of SPMS conversion. So far, however, sTREM2 has only been determined by an enzyme-linked immunosorbent assay (ELISA) test, which does not yield valid results in serum. A study of sTREM2 using the ultrasensitive Simoa method in serum or CSF has not yet been performed.

### Chitinase 3-Like 1

Chitinase 3-like 1 (CHI3L1), also known as YKL-40, is a member of the chitinase-like glycoprotein family and is predominantly produced by reactive astrocytes (174). Although its biological and physiological function in the central nervous system remains unclear, some studies have suggested that CHI3L1 is expressed in astrocytes and microglia in a variety of acute neuroinflammatory conditions, including traumatic brain injury and MS, being involved into tissue remodeling during inflammation (174–177).

CHI3L1 levels in CSF were reported to be elevated in SPMS patients and to predict SPMS conversion when NfL levels were also increased (178, 179). Interestingly, CHI3L1 levels in SPMS were similar to the level in active RRMS patients, which supports the hypothesis that inflammation remains important in the chronic phase of the disease (180). However, higher CHI3L1 levels are seen in both SPMS and PPMS patients compared to RRMS, providing a possible biomarker to differentiate between RRMS and progressive MS in general (178, 179). Accordingly, higher levels of CHI3L1 are associated with higher EDSS and related neurologic disability (181).

## Conclusion

To date, several potential clinical and paraclinical biomarkers have been researched in order to determine and predict conversion from RRMS to SPMS. The most promising clinical biomarkers are T25FW and SDMT, which evaluate both function of the lower extremities and cognition and show a good correlation with other biomarkers of MS-associated neurodegeneration. Among paraclinical biomarkers, brain and spinal cord atrophy, sNfL, GCIPL and pRNFL thinning, and decreased DI score present an easy-accessible and repeatable biomarker in determining progression of the disease. With reaching a higher degree of disability, we should aim to take more paraclinical outcome measures into consideration, especially as they show some degree of worsening even in the absence of clinical progression of the disease. However, currently available evidence for most of these biomarkers is still low.

Going forward, by conducting prospective high-quality standard studies combining multiple parameters within a multimodal approach, we could gain a more holistic view of the pathophysiology underlying SPMS conversion. Consequently, diagnostic accuracy could be improved, shortening the time to diagnosis and providing a window of opportunity for intervention to delay disability progression.

However, although a combination of the mentioned biomarkers would likely present the most sensitive tool to assess disease progression, using a large number of methods is unrealistic in everyday clinical practice. Thus, the goal would be to better define the accessible paraclinical biomarkers of conversion to SPMS, such as MRI, OCT, and biomarkers in blood, and combine the most reliable and predictive markers with clinical measurements for disease progression.

An objective and reliable definition of SPMS and a high-risk profile for SPMS conversion would enable a new approach to the management of patients with MS: DMT could be adapted or escalated in a timelier manner in order to delay or even prevent SPMS conversion, and symptomatic treatment could be intensified. In the hopefully not too distant future, these definitions might guide design and inclusion criteria when studying neuroprotective or neurorepairing agents.

## Data Availability Statement

The original contributions presented in the study are included in the article/supplementary material, further inquiries can be directed to the corresponding author/s.

## Author Contributions

NK: literature search and lead drafting the manuscript. GB: supervision of literature search and drafting of the manuscript. TB: overall supervision and review of the manuscript for intellectual content. All authors contributed to the article and approved the submitted version.

## Conflict of Interest

NK has participated in meetings sponsored by and received speaker honoraria or travel funding from Roche, Novartis, and Merck. GB has participated in meetings sponsored by, received speaker honoraria or travel funding from Biogen, Celgene, Lilly, Merck, Novartis, Roche, Sanofi-Genzyme, and Teva, and received honoraria for consulting Biogen, Celgene, Roche, and Teva. TB has participated in meetings sponsored by and received honoraria (lectures, advisory boards, consultations) from pharmaceutical companies marketing treatments for MS: Allergan, Almirall, Bayer, Biogen, Biologix, Bionorica, Celgene, MedDay, Merck, Novartis, Octapharma, Roche, Sanofi-Genzyme, Teva, and TG Pharmaceuticals. His institution has received financial support in the past 12 months by unrestricted research grants (Biogen, Merck, Novartis, Sanofi-Genzyme, Teva) and for participation in clinical trials in multiple sclerosis sponsored by Alexion, Biogen, Merck, Novartis, Octapharma, Roche, Sanofi-Genzyme, Teva.

## Publisher's Note

All claims expressed in this article are solely those of the authors and do not necessarily represent those of their affiliated organizations, or those of the publisher, the editors and the reviewers. Any product that may be evaluated in this article, or claim that may be made by its manufacturer, is not guaranteed or endorsed by the publisher.

## Abbreviations

BBSI: Brain Boundary Shift Integral
CHI3L1: chitinase 3-like 1
CSA: cross-sectional area
CSF: cerebrospinal fluid
DI: discrimination and identification
DMT: disease-modifying therapy
EDSS: Expanded Disability Status Scale
ELISA: enzyme-linked immunosorbent assay
GCIPL: ganglion cell-inner plexiform layer
GFAP: glial fibrillary acidic protein
HCLA: high-contrast visual acuity
LCLA: low-contrast letter acuity
MRI: magnetic resonance imaging
MS: multiple sclerosis
MSFC: Multiple Sclerosis Functional Composite
NEDA: no evidence of disease activity
Nf: neurofilament
NfL: neurofilament light subunit
OCT: optical coherence tomography
ON: optic neuritis
PASAT: Paced Auditory Serial Addition Test
PPMS: primary progressive multiple sclerosis
pRNFL: peripapillary retinal nerve fiber layer
RRMS: relapsing-remitting multiple sclerosis
SCA: spinal cord atrophy
SDMT: Symbol Digit Modalities Test
SD-OCT: spectral domain optical coherence tomography
SELs: slowly expanding lesions
SIENA: Structural Image Evaluation of Normalized Atrophy
sNfL: serum neurofilament light subunit
SPMS: secondary progressive multiple sclerosis
sTREM2: soluble triggering receptor 2
SWI: susceptibility-weighted imaging
TD-OCT: timed domain optical coherence tomography
TREM: triggering receptor expressed on myeloid cells 2
T25FW: Timed 25-Foot Walk Test
9HPT: 9-Hole Peg Test.
